# Adenocarcinoma Arising from Sacrococcygeal Mature Teratoma in an Adult Female: Report of a Case

**DOI:** 10.3389/fonc.2014.00117

**Published:** 2014-05-19

**Authors:** Naoki Matsumoto, Keisuke Uehara, Masataka Ando, Junki Arimoto, Takehiro Kato, Hayato Nakamura, Tomoki Ebata, Yukihiro Yokoyama, Masato Nagino

**Affiliations:** ^1^Division of Surgical Oncology, Department of Surgery, Nagoya University Graduate School of Medicine, Nagoya, Japan

**Keywords:** adenocarcinoma, teratoma, malignant transformation, sacrococcygeal

## Abstract

We report a case of adenocarcinoma arising from a sacrococcygeal mature teratoma in an adult female. A 62-year-old female was diagnosed with a presacral tumor 10 years ago. Pelvic computed tomography (CT) demonstrated a presacral heterogeneous tumor, containing multiloculated cystic area and enhanced solid component with calcification. Percutaneous needle biopsy for the solid component of the tumor identified an adenocarcinoma and the patient was diagnosed as having a sacrococcygeal teratoma with malignant transformation. Abdomino-sacral rectal resection with sacral amputation at the upper edge of the S5 was performed. The pathological diagnosis was adenocarcinoma derived from a mature teratoma. The tumor cells had infiltrated the rectal wall. After 7 months, a follow-up CT demonstrated swelling of the right inguinal lymph nodes and a right inguinal lymphadenectomy was performed. Pathological examination showed metastatic lymph nodes. The patient is doing well 21 months after the second surgery, with no signs of recurrence.

## Introduction

Teratomas are a type of germ cell tumor, which are mostly benign, and dominative in adult females (1, 2). The most common site of a primary teratoma is the ovary, followed by the testis, anterior mediastinum, retroperitoneum, and sacrococcygeal area (3–5). Sacrococcygeal teratomas are usually found in newborns or children, and can be detected prenatally; they are exceedingly rare in adults (4). Teratomas are defined as tumors, which are composed of various elements including the mesoderm, endoderm, and ectoderm. The cystic lumen mainly contains sebaceous materials and hairs. In two-thirds of cases, mature elements reflecting differentiation into normally derived cells from all three embryonic germ layers are present. However, any of these constituents has the potential to undergo malignant transformation and the incidence of such cases was reported to be 1–2% in mature ovarian teratomas (6, 7). The most common histologic type of malignant transformation in ovarian teratoma is squamous-cell carcinoma, reported in 70–80% of cases; other types of differentiation including adenocarcinoma, melanoma, and carcinoid tumor, are relatively rare (7, 8).

Although most of the patients with mature teratoma undergo surgical resection due to concern about the potential for malignant transformation, others choose to be followed-up without resection. However, early detection of malignant transformation is often difficult even with current imaging modalities, and it is unclear when the malignant transformation occurs during the follow-up period. We report a case of adenocarcinoma arising from a sacrococcygeal mature teratoma in an adult female who had been followed-up for 10 years.

## Case Report

**Figure 1:**
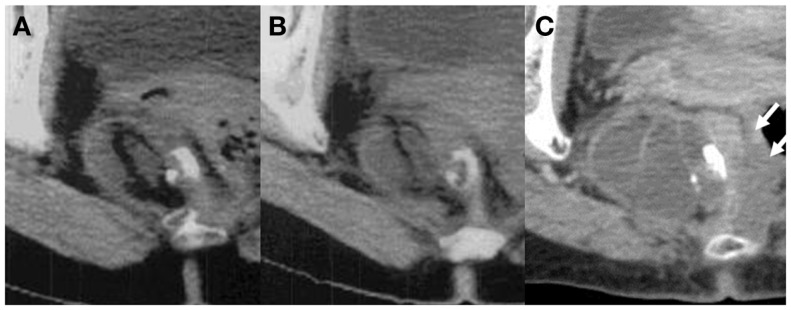
**Longitudinal change of the sacrococcygeal mature teratoma: (A) 10 years before surgery (45.4 mm × 35.9 mm), (B) 5 years before surgery (49.1 mm × 35.4 mm), and (C) just before surgery (67.2 mm × 45.4 mm)**. The tumor size and shape did not change in the first 5 years, however, in the last 5 years, the size of the tumor increased 1.4-fold and the solid component invaded the rectal wall (white arrows).

**Figure 2:**
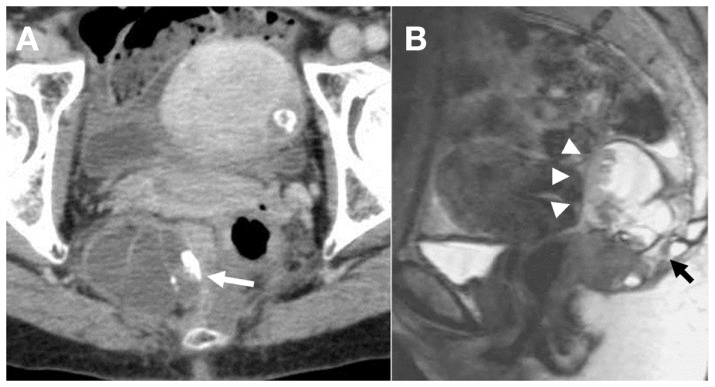
**(A)** Enhanced CT shows a presacral heterogeneous tumor, 67 mm × 45 mm, containing multiloculated cystic area and solid component with calcification (white arrow). **(B)** T2-weighted sagittal magnetic resonance image demonstrates a presacral non-enhanced cystic lesion with solid component, which was suspected to be strongly adhered to the rectal wall (white arrow heads), S5, and coccyx (black arrows).

A 62-year-old female who presented with anal pain for 3 months was referred to our hospital. Ten years at another previous hospital she was diagnosed as having both myoma of the uterus and a presacral tumor on computed tomography (CT). Five years later, a repeat pelvic CT scan showed no change in shape or size of the presacral tumor (Figure 1). On admission to our hospital, digital examination showed a palpable hard mass with smooth surface beyond the right-posterior wall of the rectum. Routine laboratory tests were all within normal ranges. The serum carcinoembryonic antigen (CEA) was 2.0 ng/ml (normal range, <5 ng/ml) and carbohydrate antigen 19-9 (CA19-9) was 434 U/ml (normal range, <37 U/ml). An enhanced pelvic CT scan demonstrated a presacral heterogeneous tumor, 67 mm × 45 mm, containing a multiloculated cystic area and enhanced solid component with calcification. There was no evidence of distant metastasis. Magnetic resonance imaging (MRI) showed a presacral non-enhanced cystic lesion with solid component, which was suspected of having invaded the rectal wall, fifth sacral vertebra (S5), and coccyx (Figure 2). Percutaneous needle biopsy of the solid component of the tumor indicated an adenocarcinoma. Based on these findings, the patient was diagnosed as having a sacrococcygeal teratoma with malignant transformation into adenocarcinoma.

**Figure 3:**
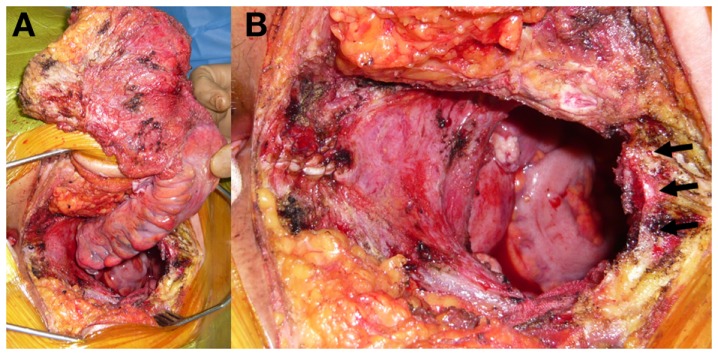
**(A)** Intraoperative photographs show *en-bloc* resection of the tumor with the rectum, fifth sacral vertebra and coccyx. **(B)** Black arrows show the cut surface of upper edge of the S5.

Abdomino-sacral rectal resection with permanent colostomy was performed. On laparotomy, there was no evidence of liver metastasis or peritoneal dissemination. After the large uterine myoma was raised, the tumor was palpable on the right side of the rectum. The uterine myoma was not removed. The rectum was fully mobilized and the periosteum of the upper edge of the S5 was exposed. The second step was sacral resection. The patient was placed in a jackknife position, a median incision including the anus was made, and a sacral amputation was carried out using the chisel and hammer at the upper edge of the S5. Following this, the tumor was completely resected from the pelvic floor (Figure 3).

**Figure 4:**
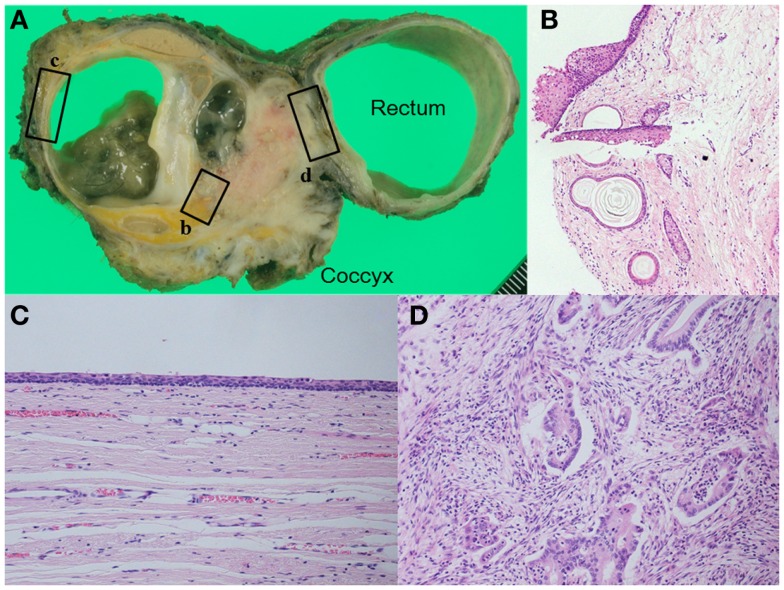
**(A)** Macroscopic finding shows a complex tumor consisting of multilocular cyst and solid component. The cyst includes sebaceous debris with hairs. Pathological examination shows **(B)** hair follicles and sebaceous follicles in the cystic wall, **(C)** the wall of the cyst was lined by ciliated respiratory type epithelium, and **(D)** adenocarcinoma had invaded the rectal wall. [**(B)**: HE, ×100, **(C,D)**: HE, ×200].

Macroscopically, the tumor was a multilocular cystic lesion containing sebaceous matter, hair, and follicles (Figure 4A). The pathological diagnosis was an adenocarcinoma derived from a mature teratoma (Figures 4B–D). There were no lymph node metastases but there was evidence of a slight lymphatic invasion around the rectal wall. The tumor cells had infiltrated into the muscular layer of the rectum. A small number of cancerous cells were present at the radial margin behind the vaginal wall, and the final pathological diagnosis was judged to be an R1 resection (the margins of the resected parts show tumor cells when viewed microscopically). The patient developed a pelvic sepsis but it was treated by conventional open drainage and use of antibiotics, and the postoperative hospital stay was 19 days. The patient did not receive any adjuvant therapy.

**Figure 5:**
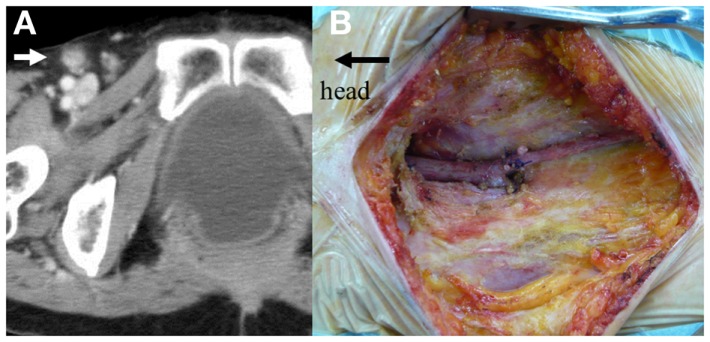
**(A)** Pelvic computed tomography reveals swelling of the right inguinal lymph nodes (white arrow). **(B)** Intraoperative photograph after right inguinal lymphadenectomy.

After 7 months, a follow-up CT demonstrated swelling of the right inguinal lymph nodes (Figure 5A). The additional 18F-fluorodeoxyglucose positron emission tomography/CT showed that the maximum standardized uptake value was 9.6, and the swollen lymph nodes were judged to be metastatic lesions. There was no evidence of distant metastasis or local recurrence. A right inguinal lymphadenectomy was performed (Figure 5B). Pathological examination showed adenocarcinoma in 3 of 12 harvested lymph nodes. The patient is doing well 21 months after the second surgery, with no signs of recurrence.

## Discussion

It is generally accepted that the patients with sacrococcygeal mature teratomas are suitable for surgical treatment because of a malignant potential. Once the malignant transformation arises, only en-bloc radical resection offers the possibility of a good outcome. However, it is often difficult to achieve R0 resection when the tumor has become large or has invaded surrounding organs in the narrow pelvis. Therefore, various surgical approaches have been reported, including trans-abdominal, trans-sacral, and a combined abdomino-sacral approach (9–11). In our case, an abdomino-sacral approach was adopted because of the suspicion that the tumor had invaded, not only the rectum but also the distal sacrum and coccyx. The surgical approach selected should be based on various factors including tumor size, location, and invasion to the surrounding organs, and appropriate selection might be the key to successful operation.

Unfortunately, the radial margin was microscopically positive (R1) in the present case. Although adjuvant chemotherapy, radiotherapy, and surgical re-resection were all treatment options, we did not administer any additional therapy. Several studies have investigated the efficacy of adjuvant chemotherapy and have shown that systemic therapy was beneficial to few patients with malignant transformation from a teratoma. However, the majority of these results were related to the efficacy of adjuvant chemotherapy in squamous-cell carcinomas arising from ovarian teratoma (12, 13). Whereas, radiotherapy has been reported to be a promising approach, whole pelvic radiotherapy increases the risk of late complications such as sexual and voiding dysfunctions, intestinal and defecation problems, and secondary carcinogenesis (14–16). The best adjuvant therapy for this disease has not been defined and we selected to follow-up without any additional treatment.

The pathway from the perineal cutaneous part of the anal canal and the vulva to superficial inguinal nodes is recognized as the “superficial perineal accessory pelvic pathway” (17). In cases with rectal adenocarcinomas invading the dentate line, inguinal lymph node recurrence sometimes develops even after curative resection, although the prognosis is likely to be poor (18). In our case, histologic findings at the initial operation demonstrated that the tumor had invaded the muscular layer of the low rectum and lymphatic invasion was observed. We elected to perform an inguinal lymphadenectomy without radiotherapy and the patient is doing well after 21 months of follow-up, with no signs of recurrence.

In the majority of cases, when a CT and/or MRI scan reveals a sacrococcygeal teratoma, surgical resection is immediately selected because of concerns over malignant transformation. However, it is still unclear when the malignant transformation happens in a benign tumor and how we should observe patients who elect not to have surgical resection. A unique feature of the present case is that the patient had been followed-up by CT examination for 10 years at a previous hospital. The tumor size and shape did not change in the first 5 years, however, in the second 5-year period, there was a 1.4-fold increase in the size of the tumor and the solid component invaded the rectal wall. Although, clearly, an early diagnosis of malignant transformation offers the best hope of a successful outcome, it is also the case that once malignant transformation occurs, localized involvement and metastases can develop rapidly in some cases (19). In cases where non-surgical treatment is decided upon, fairly frequent follow-up is recommended.

Another debatable point is whether preoperative needle biopsy is essential. Percutaneous needle biopsy is crucial for an accurate diagnosis and it might allow for a consensus decision in the subsequent treatment of patients with germ cell tumors (20). However, this procedure is associated with risk at the point of implantation or provoking dissemination when the tumor has malignant behavior. In the present case, we resected the route of biopsy completely to prevent postoperative tract recurrence.

In conclusion, we describe an extremely rare case of a patient with a sacrococcygeal mature teratoma with malignant transformation into an adenocarcinoma. Surgical resection was effective for both primary and metastatic inguinal node disease. In case of patients with mature teratoma who opt for non-surgical treatment, fairly frequent follow-up is recommended.

## Conflict of Interest Statement

The authors declare that the research was conducted in the absence of any commercial or financial relationships that could be construed as a potential conflict of interest.
